# Recent insights into the structure and function of Mitofusins in mitochondrial fusion

**DOI:** 10.12688/f1000research.16629.1

**Published:** 2018-12-28

**Authors:** Mickael M Cohen, David Tareste

**Affiliations:** 1 Sorbonne Université, CNRS UMR8226, Institut de Biologie Physico-Chimique, Laboratoire de Biologie Moléculaire et Cellulaire des Eucaryotes, Paris, France; 2 Université Paris Descartes, Sorbonne Paris Cité, INSERM ERL U950, Trafic Membranaire dans le Cerveau Normal et Pathologique, Paris, France; 3 Université Paris Descartes, Sorbonne Paris Cité, INSERM UMR 894, Institut de Psychiatrie et Neurosciences de Paris, Paris, France

**Keywords:** Mitochondria, Membrane, Fusion, SNARE, Hemagglutinin, Atlastin, Mitofusin, GTPase, Heptad Repeat, Coiled-coil, Amphipathic Helix, Lipids

## Abstract

Mitochondria undergo frequent fusion and fission events to adapt their morphology to cellular needs. Homotypic docking and fusion of outer mitochondrial membranes are controlled by Mitofusins, a set of large membrane-anchored GTPase proteins belonging to the dynamin superfamily. Mitofusins include, in addition to their GTPase and transmembrane domains, two heptad repeat domains, HR1 and HR2. All four regions are crucial for Mitofusin function, but their precise contribution to mitochondrial docking and fusion events has remained elusive until very recently. In this commentary, we first give an overview of the established strategies employed by various protein machineries distinct from Mitofusins to mediate membrane fusion. We then present recent structure–function data on Mitofusins that provide important novel insights into their mode of action in mitochondrial fusion.

## Introduction

Mitochondria are delimited by an outer membrane and include an inner membrane that separates a matrix and an intermembrane space. *In vivo*, these double-membraned organelles assemble as a network that continually changes shape through fusion, fission, and mobility along cytoskeleton tracks. The ability of mitochondria to fuse and divide is intricately tied to the physiological context of a cell. For instance, during mitosis in mammalian cells or meiosis in yeast cells, the mitochondrial network fragments to ensure appropriate mitochondrial partitioning between mother and daughter cells before reassembling through fusion ^1, 2^. Similarly, when mitochondrial activity needs to be increased at precise cellular locations, tubules separate from the main network, migrate through the cytosol, and fuse with pre-existing mitochondria on the target site ^3^. Mitochondrial fusion and fission also have specific functions linked to the quality control and functional efficiency of the organelle ^4^. Fusion promotes homogenization of mitochondrial contents and favors functional complementation between mitochondria with distinct lesions in their nucleoids or proteome. Conversely, fission allows the segregation of non-functional mitochondria and their subsequent degradation by autophagy. Since fusion and fission are essential for proper mitochondrial homeostasis and function, their alteration leads to numerous pathologies including severe neurodegenerative disorders ^5– 7^.

While mitochondrial dynamics have been observed in many organisms since the beginning of the 20 ^th^ century ^8^, the machineries that govern mitochondrial fusion and fission remained mysterious until the late 90s and the beginning of the 21 ^st^ century (1997–2004). The first identified fusion factor was a large GTPase from the dynamin-related protein (DRP) superfamily ^9^. Male Drosophila with mutations in the gene encoding for this protein were sterile and exhibited fuzzy onion-like mitochondria in early post-meiotic spermatids. This phenotype resulted from an inhibition of mitochondrial fusion, thus blocking the formation of giant mitochondria known as the Nebenkern. This first factor found to be involved in mitochondrial fusion was logically called Fuzzy Onion (Fzo). Shortly after this landmark study, Fzo1, the yeast homologue of Fzo, was shown to promote mitochondrial fusion ^10, 11^. Two mammalian homologues of Fzo, Mitofusin 1 (MFN1) and Mitofusin 2 (MFN2), were then identified ^12– 17^. Then came the year 2004, which was exceptional in terms of discoveries in Mitofusin function. Mitofusins were shown to promote mitochondrial docking through their auto-oligomerization in trans (from adjacent outer membranes) ^18^, and the setup of an *in vitro* mitochondrial fusion assay provided the first demonstration that outer and inner membrane fusion were separable events but also that Fzo1 was specifically involved in the fusion of outer membranes ^19^.

This 1997–2004 pioneering period for mitochondrial dynamics was not limited to the discovery of Mitofusins. A second set of large transmembrane GTPases from the DRP family, the yeast Mgm1 and its mammalian homologue OPA1, was found to reside in the inner mitochondrial membrane and to mediate inner membrane fusion ^20– 22^. A third set of DRPs, located in the cytosol, the yeast Dnm1 and its mammalian homologue DRP1, was shown to induce mitochondrial fission ^23– 25^. DRP1 was found to self-assemble into ring- and spiral-like structures that could fit the size of mitochondrial constriction sites ^25, 26^. Further mechanistic investigations identified mitochondrial recruiters for Dnm1 and DRP1 ^27– 32^, revealed deep structural insights on their constriction mechanisms of mitochondrial tubules ^33– 35^, and demonstrated that endoplasmic reticulum (ER) wrapping around mitochondrial tubules was a pre-requisite for the recruitment of the fission apparatus ^36^.

Mechanistic inputs on core mitochondrial fusion machineries, on the other hand, faced a significant roadblock with the challenge of purifying recombinant Mitofusins/Fzo and OPA1/Mgm1. Nonetheless, structural analysis of a short recombinant form of Mgm1 lacking its transmembrane domain (TMD) ^37– 40^ and biochemical characterization of purified full-length Mgm1 and OPA1 ^38, 41^ have provided significant mechanistic insights on inner membrane fusion. In contrast, full-length Mitofusins/Fzo have not yet been successfully isolated. However, their possible mode of action can, at this point, be inferred from numerous experimental observations accumulated during the last two decades and the comparison with other well-characterized fusion systems, such as SNARE and viral fusion proteins, or other fusion DRPs. In this review, we first present the core molecular mechanisms of previously described fusion machineries and then discuss recent biochemical, biophysical, and structural data on Mitofusins that suggest how they might function in mitochondrial fusion.

## Overview of cellular membrane fusion machineries

Membrane fusion reactions require specialized proteins whose structure and function have evolved to help membranes advance through the successive energy-demanding intermediate stages of fusion ^42^. These stages include (i) membrane recognition and docking, (ii) membrane approach and deformation, (iii) membrane destabilization and merging (with the potential formation of a hemifused structure, where the outer leaflets of the lipid bilayers have merged, while their inner leaflets and aqueous compartments remain separated), and (iv) formation and growth of a fusion pore, leading to mixing of the two aqueous compartments. The molecular architecture of fusion proteins is often divided into several functional domains that can orchestrate one or more of these intermediate stages leading to fusion. Membrane fusion events are also regulated by additional molecular factors (lipids or proteins) that have the capacity to modify the structure and function of fusion proteins and/or lipid bilayers to make the fusion reaction energetically more favorable ^43– 45^.

During the last two decades, significant progress has been made toward identifying the key molecular players and underlying biophysical mechanisms of membrane fusion machineries. The soluble N-ethylmaleimide-sensitive factor attachment protein receptor (SNARE) proteins of neurotransmission and the hemagglutinin (HA) protein of the influenza virus have notably been the subject of many structural and functional studies, allowing a precise characterization of their mode of action ^45– 48^. Both machineries use the energy released during the formation of a coiled-coil complex of α-helices to drive membrane fusion.

During neurotransmission, synaptic vesicles are first docked to the pre-synaptic plasma membrane by Rab GTPase proteins and tethering factors. At this stage, cognate membranes are still reversibly linked and separated by tens of nanometers ^49^. Membranes are next stably docked to each other by the formation of a membrane-bridging four-helix coiled-coil trans-SNARE complex composed of the cytosolic heptad repeat (HR) domains of the synaptic vesicle (v-) SNARE protein and the target plasma membrane (t-) SNARE protein ^50, 51^. This trans-SNARE complex assembles like a zipper, from its N-terminal (membrane-distal) region toward its C-terminal (membrane-proximal) region, bringing cognate membranes in close apposition as it folds up ^52– 54^. Membrane fusion occurs as a result of lipid bilayer proximity, local deformation, and rupture as SNARE zippering progresses all the way into the membrane through the assembly of v- and t-SNARE TMDs ^55– 57^.

In the case of influenza virus infection, the viral envelope membrane is docked to the host cell membrane when the N-terminal amphipathic fusion peptide of the HA protein is released from its hydrophobic pocket within the HA molecule and inserts into the target membrane. The two membranes are next brought in close apposition when the HA protein folds back on itself in the form of a six-helix coiled-coil complex, pulling together the HA fusion peptide in the target membrane and the HA TMD in the envelope membrane ^58^. Owing to its amphipathic character, the HA fusion peptide is also believed to destabilize the target membrane by perturbing lipid bilayer packing and/or inducing high local curvature ^59^. Thus, HA-mediated fusion occurs as a result of a “jack-knife” self-folding mechanism to bring membranes into close proximity and lipid bilayer destabilization by the amphipathic HA fusion peptide.

Another mechanism for fusion, which does not use the folding energy of coiled-coil structures, has been described in the case of the homotypic fusion of ER tubules by the large membrane-anchored DRP Atlastin (ATL) of the ER membrane ^60^. The structure of ATL consists of an N-terminal GTPase domain, a three-helix bundle middle domain, two TMDs, and a C-terminal cytoplasmic tail. ER membrane fusion starts with the formation of a loosely docked state involving trans-ATL complexes that interact via their GTPase domain. GTP hydrolysis then triggers membrane approach through the development of a tightly docked state stabilized by interactions involving both the GTPase and the middle domains of ATL ^61– 63^. This conformational change releases the C-terminal tail of ATL that contains an amphipathic helix with the capacity to bind and perturb the lipid bilayer structure ^64^. The fusion of ER tubules thus proceeds as a result of ER membrane approach by GTP-dependent conformational rearrangements of membrane-bridging trans-ATL complexes and ER membrane destabilization by the C-terminal amphipathic helix of ATL ^65– 67^.

Two bacterial DRPs, BDLP1 and DynA, were also involved in membrane fusion reactions. BDLP1 might regulate thylakoid morphology in the cyanobacteria *Nostoc punctiforme* ^68^, whereas DynA has been implicated in the maintenance of cell membrane integrity in the Gram-positive bacteria *Bacillus subtilis* ^69, 70^. BDLP1 is composed of a GTPase domain, two four-helix bundle domains (the membrane-distal neck region and the membrane-proximal trunk region), and a paddle region that allows transient membrane anchoring, whereas DynA consists of two fused DRPs, D1 and D2, where only D1 possesses a paddle region. X-ray and cryo-electron microscopy analysis of BDLP1 revealed two distinct conformations depending on the nucleotide-binding state of the protein. In its nucleotide-free or GDP-bound state, BDLP1 was crystallized as a bent dimeric conformation mainly stabilized by an interaction between the GTPase domains but also involving contacts between the trunk and the paddle regions of two adjacent molecules ^68^. When bound to a non-hydrolyzable GTP analogue, BDLP1 was shown to transit to an extended conformation that still involved dimerization of the GTPase domain and could further polymerize into a helical structure on the membrane of liposomes ^71^. Such macromolecular assembly, by acting together with membrane insertion of the paddle region, induced liposome tubulation. It was thus proposed that GTP hydrolysis might trigger membrane detachment and disassembly of the BDLP1 helical polymer, leading to protein-free membrane tubules of high curvature that must fuse into larger structures to relieve their elastic stress. Contrary to BDLP1, DynA was shown to bind and assemble onto liposomes in the absence of nucleotide ^72^. DynA also induced liposome docking and fusion, independently of GTP, through a mechanism that required only the presence of magnesium ions. In addition, DynA removal from the liposome membrane by proteolysis led to the formation of larger fused liposomes, echoing the hypothesis that depolymerization of BDLP1 from membrane tubules could trigger their fusion.

## Main properties and molecular architecture of Mitofusins

**Figure 1.**
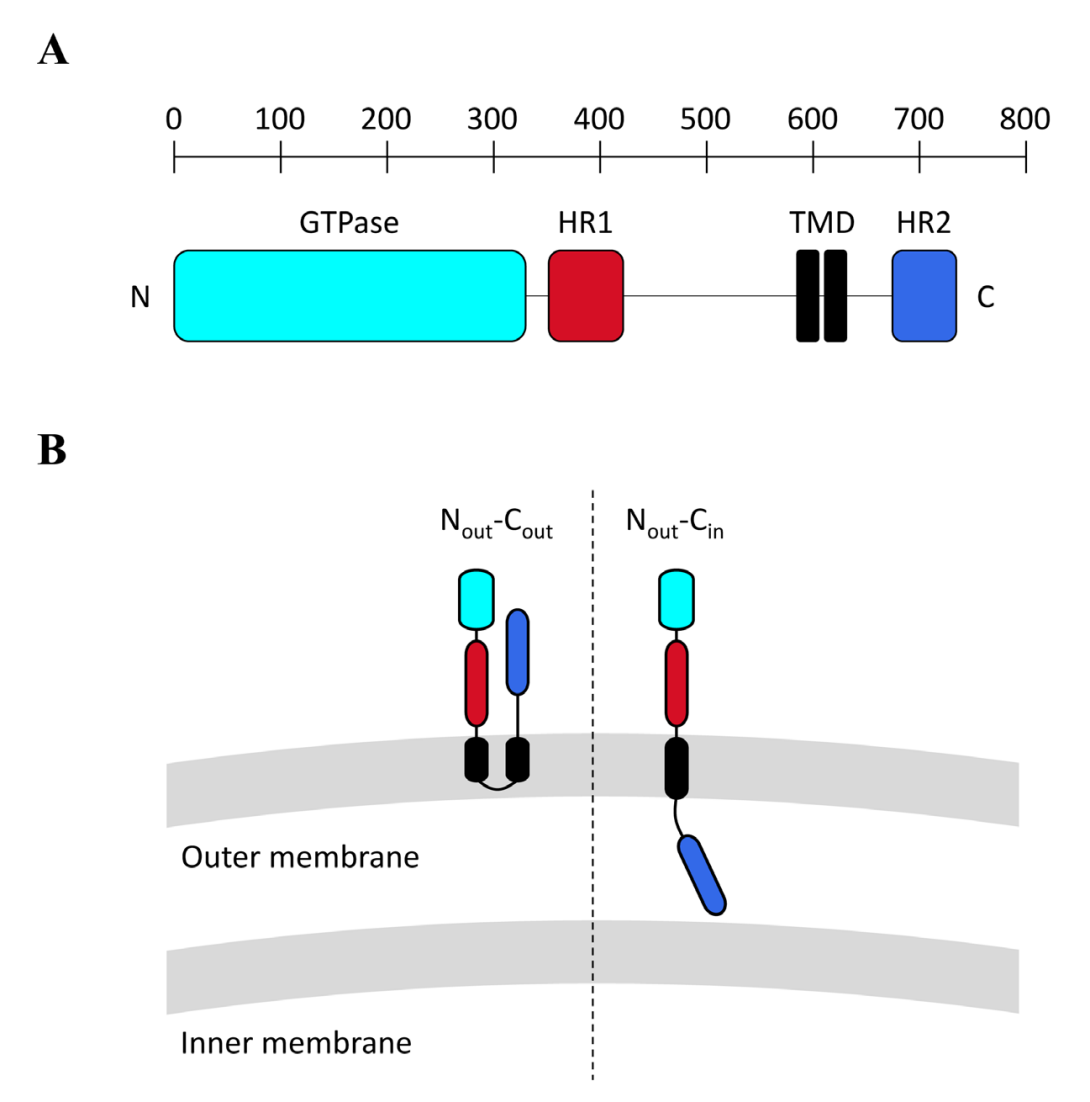
( **A**) Molecular architecture of Mitofusin 1 (MFN1). Like MFN1, all Mitofusins include an N-terminal GTPase domain (light-blue) and two C-terminal heptad repeat domains, HR1 (red) and HR2 (dark blue), that sandwich a transmembrane region (black). The yeast Mitofusin Fzo1 includes an additional heptad repeat domain (HRN) located upstream of the GTPase domain (not depicted here). ( **B**) Possible topologies of Mitofusins. (Left) A transmembrane region with two transmembrane domains (TMDs) gives Mitofusins a topology in which the N- and C-terminal extremities are exposed to the cytoplasm (N _out_–C _out_ topology). (Right) It was recently demonstrated that Mitofusins from vertebrates could also include a single TMD, which keeps the N-terminal GTPase and HR1 domains in the cytoplasm but places the C-terminal HR2 domain in the mitochondrial intermembrane space (N _out_–C _in_ topology). Note that the BDLP1-like folding of Mitofusins observed in the X-ray structures of MFN1 ( Figure 2) is compatible with the N _out_–C _out_ but not the N _out_–C _in_ topology.

The primary sequence of all Mitofusins from fungi (Fzo1), Drosophila (Fzo and Marf), or vertebrates (MFN1 and MFN2) is characterized by an N-terminal GTPase domain and two C-terminal HR1 and HR2 domains that surround a transmembrane region. In yeast, an additional HR domain (HRN) is located N-terminal of the GTPase domain ( Figure 1A). The integrity of all of these domains is essential for Mitofusin function in mitochondrial fusion ^10, 12, 14, 15, 18, 73– 75^.

It is established that the transmembrane region of Fzo1 is constituted of two distinct TMDs that allow insertion of the yeast Mitofusin in outer membranes and exposure of both HR1 and HR2 in the cytoplasm ( Figure 1B, left) ^76^. Early observations indicated that Mitofusins from vertebrates displayed a similar topology ^14^. However, this view was recently challenged with phylogenetic analysis suggesting that Mitofusins from vertebrates, Drosophila, or nematodes may harbor a single TMD in between HR1 and HR2 ( Figure 1B, right) ^77^. Such domain organization was observed in this study for human MFN1 and MFN2, which were found to expose their HR2 in the intermembrane space and their HR1 in the cytoplasm. Nonetheless, it was also demonstrated that C-terminal tagging of MFN2 could force the exposure of HR2 in the cytoplasm and that this N _out_–C _out_ topology was competent to rescue mitochondrial fusion partially but significantly in MFN2 knockout cells. It is thus tempting to speculate that Mitofusins from vertebrates, Drosophila, and nematodes could adopt two distinct N _out_–C _in_ or N _out_–C _out_ topologies. The requirement for two Mitofusin topologies in metazoans may serve specific functions, such as the coordination between outer and inner membrane fusion. In yeast, this function involves Ugo1, a protein of the outer mitochondrial membrane that physically interacts with both Fzo1 and Mgm1 ^78, 79^ and is essential for Fzo1- and Mgm1-mediated fusion of outer and inner membranes, respectively ^80^. SLC25A46, the mammalian homologue of Ugo1, also interacts with both MFN2 and OPA1 ^81^. However, knockdown of SLC25A46 was shown to increase mitochondrial fusion ^82^, suggesting that the role for Ugo1 in mitochondrial fusion is not conserved from yeast to metazoans. In this context, one could speculate that N _out_–C _in_ Mitofusins might function as co-factors for N _out_–C _out_ Mitofusins, like Ugo1 for Fzo1.

Regardless of Mitofusins’ topology in the outer mitochondrial membrane, the primary function of all Mitofusins is to promote docking and fusion of mitochondria. The docking activity involves a recurrent feature of DRPs, which is to self-assemble into oligomers. Consistent with this, Mitofusins induce mitochondrial docking by auto-oligomerizing in both cis (i.e. within the same membrane) and trans (i.e. across two opposing membranes) ^11, 16, 18, 76, 83– 85^. While the mechanisms of trans-oligomerization are emerging (see below), those of cis-oligomerization are currently lacking any structural insights. Yet cis-oligomerization of Mitofusins was shown to require some co-factors and post-translational modifications. In yeast, Ugo1 was found to favor cis-dimerization of Fzo1 ^85^. In mammals, disulfide bridges were shown to trigger cis-oligomerization of MFNs, which stimulated mitochondrial fusion ^77, 86^.

Another important feature of Mitofusins is that they can be found in locations other than on the outer mitochondrial membrane. MFN2 from metazoans also localizes on ER membranes, where it regulates ER stress ^87, 88^ as well as contacts between ER and mitochondria ^89^, either positively ^90, 91^ or negatively ^92– 94^. Similarly, Fzo1 was recently found to co-localize with peroxisomes and to promote peroxisome–mitochondria contacts ^95^. Mitofusin-mediated contacts between mitochondria and ER, or mitochondria and peroxisomes, may involve trans-oligomerization mechanisms. While this remains to be proven for Fzo1 in peroxisome–mitochondria contacts, the pool of MFN2 on ER membranes was shown to interact with the mitochondrial pools of both MFN1 and MFN2 ^89^. This raises a fundamental question: how can Mitofusin trans-oligomers mediate ER–mitochondria contacts without promoting heterotypic fusion between ER and outer mitochondrial membranes? One possibility is that the capacity of Mitofusins to promote mitochondrial fusion is intimately linked to the composition of the outer mitochondrial membrane, in which specific lipids ^98– 100^ or protein co-factors, such as Ugo1 or the N _out_–C _in_ Mitofusins ^77, 101– 105^, could modulate the oligomerization, mobility, and/or fusogenic properties of Mitofusins.

## Mechanisms for mitochondrial docking

First insights into the molecular mechanisms underlying mitochondrial docking were obtained with the crystallization of the HR2 domain of MFN1 ^18^. In this work, HR2 was shown to form a 9.5 nm long homodimeric coiled-coil complex of two α-helices arranged in an antiparallel configuration. In addition, *in situ* expression of MFN1 variants lacking their GTPase domain induced the accumulation of docked mitochondria separated by a uniform gap of ~16 nm, and the effect was abolished when these truncated MFN1 variants were carrying mutations destabilizing the HR2 coiled-coil structure. Based on these observations, it was thus proposed that HR2 might act at the docking stage during mitochondrial fusion. In line with this, the isolated HR2 domain of MFN1 was shown to mediate liposome docking *in vitro* ^106^, and soluble HR1 fragments of MFN2 competing with intramolecular HR1/HR2 interaction ^14, 75, 107^ were hypothesized to expose HR2, allowing the development of a docking-competent MFN2 conformation ^108^.

**Figure 2.**
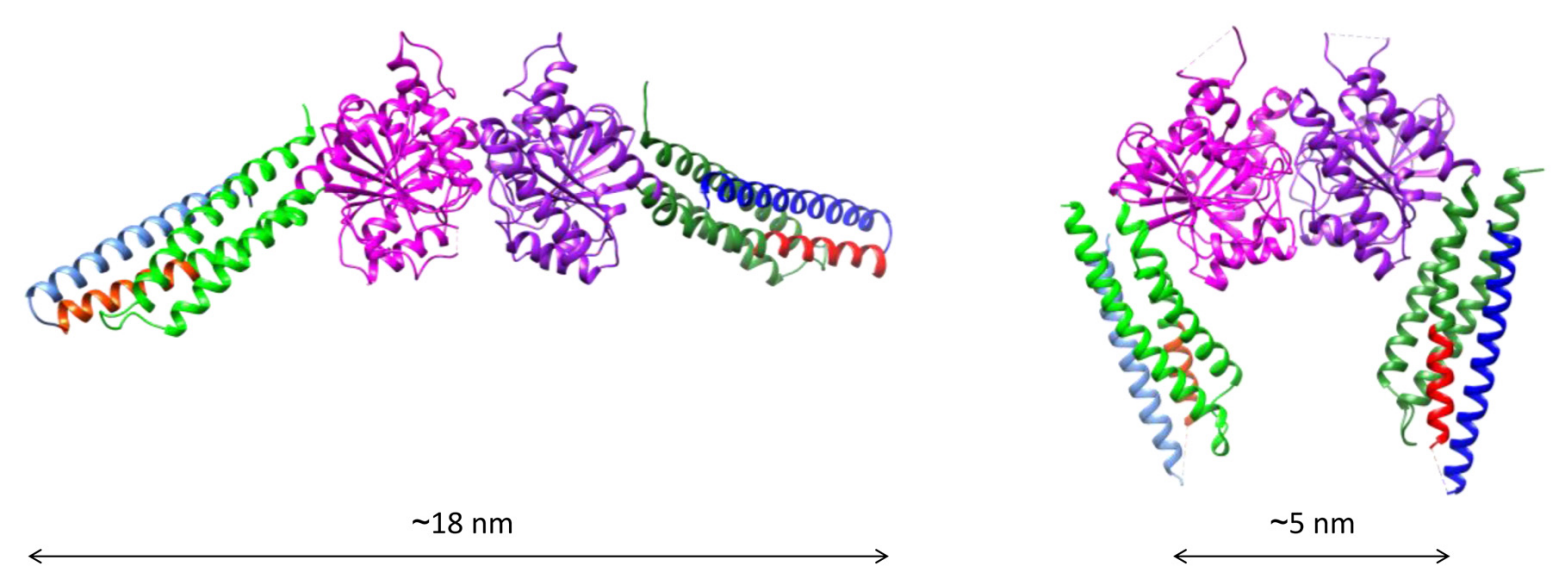
X-ray structure of a Mitofusin 1 (MFN1) fragment. The fragment is composed of the predicted GTPase domain (purple and green) and the first ~15 N-terminal residues of the heptad repeat domain HR1 (red) linked to the last ~45 C-terminal residues of the HR2 domain (blue) via an artificial linker. The structure of this fragment, named mini-MFN1, consists of a typical G-domain (purple) and a four-helix bundle domain (HB1), which includes two helices from an N-terminal extension of the GTPase (green), the short N-terminal fragment of HR1 (red), and the C-terminal fragment of HR2 (blue). (Left) Structure of the “open-HB1” dimeric form of mini-MFN1 (Protein Data Bank entry 5GOM ^96^) obtained upon addition of GDP/AlF _4_^–^ (but with only GDP in the crystal). (Right) Structure of the “closed-HB1” dimeric form of mini-MFN1 (Protein Data Bank entry 5YEW ^97^) observed in the presence of GDP/BeF _3_^–^. The indicated distances were measured between the N-terminal sides of HR2. The figures were prepared using Chimera.

**Figure 3.**
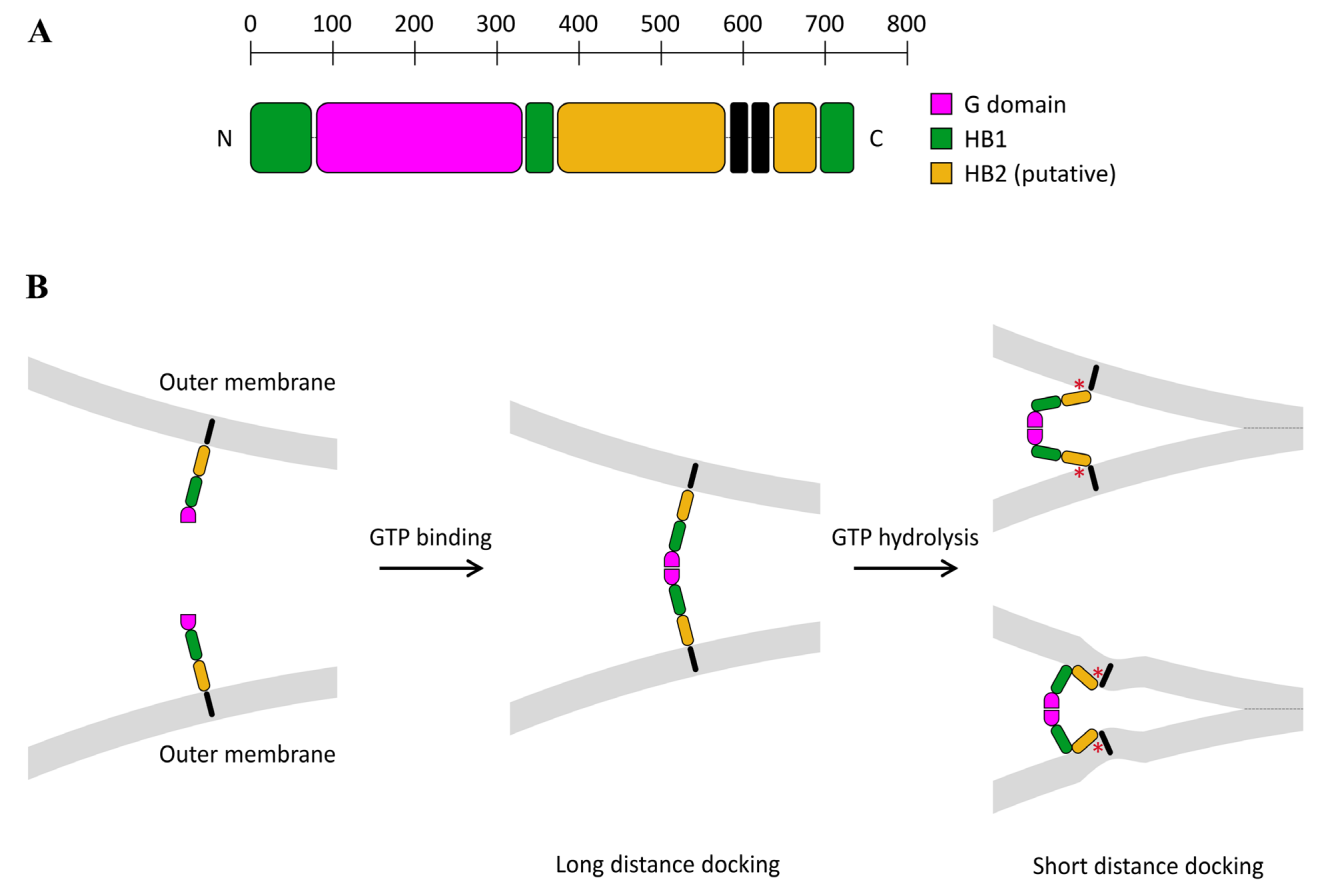
Hypothetical mode of action of Mitofusin in mitochondrial fusion. ( **A**) Based on the available X-ray structures of Mitofusin 1 (MFN1) ( Figure 2) ^96, 97, 109^, as well as structural modeling of MFN1 and Fzo1 using BDLP1 as a template ^96, 110^, full-length Mitofusins should be constituted of four distinct structural motifs in their three-dimensional conformation: a G-domain (pink) followed by two sequential four-helix bundles, HB1 (green) and HB2 (yellow), and a transmembrane region that spans the outer membrane twice. ( **B**) Mitofusin molecules may dimerize across outer mitochondrial membranes upon GTP binding, which leads to long-distance (~20 nm) docking of mitochondria. GTP hydrolysis may then induce a large conformational rearrangement of Mitofusin, through either a “scissor-like” (top panel) or a “self-folding” (bottom panel) mechanism, which brings outer mitochondrial membranes in closer proximity (short-distance docking). These two docking states may be further stabilized by the formation of a ring of trans-Mitofusin complexes (not depicted here) at the periphery of the contact zone between mitochondria. Short-distance docking may also be reinforced by the formation of antiparallel trans-HR2 dimers (not shown for clarity). Mitochondrial fusion may proceed as a result of local membrane deformation near the TMD when Mitofusin undergoes its GTP hydrolysis-dependent conformational transition and membrane structure perturbation by the HR1 domain (symbolized by the red star).

Three recent X-ray analyses of a mini-MFN1 construct consisting of the predicted N-terminal GTPase domain linked to the second half of the C-terminal HR2 domain suggested an alternative mechanism for mitochondrial docking ^96, 97, 109^. This mini-MFN1 was found to dimerize in solution in the presence of GTP or the transition state analogues GDP/BeF _3_^–^ or GDP/AlF _4_^–^. The dimers could be crystallized only in the presence of the transition state analogues and displayed two alternative configurations ( Figure 2): an open conformation obtained upon the addition of GDP/AlF _4_^–^ but which only displayed GDP in the crystal ^96^ and a closed conformation obtained with GDP/BeF _3_^–^^97^. In both configurations, the mini-MFN1 featured a typical G-domain followed by a four-helix bundle (HB1, which included three helices from the GTPase domain and one helix from the HR2 domain) and dimerized via its G-domain with the nucleotide-binding pockets facing each other. In the open form ( Figure 2, left), the HB1s were found to run perpendicular to the G-domain interface and to point in opposite directions, whereas in the closed form ( Figure 2, right), they were parallel to the G-domain interface and were pointing in the same direction. Importantly, the “closed-HB1” dimer was found by fluorescence resonance energy transfer (FRET) measurements to be stronger than the “open-HB1” dimer ^97^. Interestingly, ATL was also shown to dimerize in the presence of GTP and to undergo an open–closed conformational transition of its three-helix bundle middle domain upon GTP hydrolysis, leading to a tighter ATL dimer ^60, 63^. Similar to ATL, it is thus possible that Mitofusin forms a homotypic membrane-bridging complex upon GTP binding and brings membranes in close apposition through a GTP-dependent conformational change ( Figure 3B).

It is worth noting that these two possible mechanisms for mitochondrial docking are not mutually exclusive, since GTPase-mediated docking could occur first at long distance, to bring membranes from a few tens of nanometers down to a few nanometers apart, and trans-HR2 dimers could next stabilize docking at short distances. This scenario would be comparable to the sequential action of Rab GTPases and the HR domain of SNAREs in intracellular vesicle docking events (with the exception that HR2 cannot bring membranes in molecular proximity to induce fusion). The exact nature of the conformational transition of Mitofusin leading to mitochondrial docking will require the structural characterization of the membrane-proximal region of the protein. However, some clues can be found in the structural analogy between Mitofusins and BDLP1. The structure of mini-MFN1 is in fact identical to that of the GTPase and neck regions of BDLP1. Secondary structure prediction of MFN1 and computational modeling of full-length Fzo1 using BDLP1 as a template suggest that the membrane-proximal region of Mitofusins might form a second four-helix bundle (HB2, including HR1, two other helices preceding the TMD, and the first half of HR2; Figure 3A), which would be similar to the trunk region of BDLP1 ^109, 110^. If one assumes helical continuity between the two HBs, Mitofusin could mediate membrane approach through a GTP hydrolysis-dependent scissor-like mechanism ^97^ ( Figure 3B, top right). If, like BDLP1, bending occurs between the two HBs following GTP hydrolysis ^71^, Mitofusin could bring membranes in close proximity by folding back on itself ( Figure 3B, bottom right).

The energy generated by the large conformational changes described here for Mitofusin is unknown, but it is expected that the synergistic action of several Mitofusin dimers will be required to bring mitochondrial membranes in close apposition. This possibility is consistent with the recent discovery of a Mitofusin docking ring complex ^111^. By combining the power of the *in vitro* mitochondrial fusion assay ^19^ with that of cryo-electron tomography analysis, the docking and outer membrane fusion of yeast mitochondria were found to take place through distinct substages ^111^. Isolated mitochondria were shown to first undergo long-distance docking by protein densities accumulating in between outer membranes. In this state, outer membranes were never closer than 7–8 nm apart and the protein densities often arranged in regular repetitions of globular structures, likely corresponding to Fzo1 oligomers. Importantly, GTP hydrolysis was shown to progressively bring outer membranes closer together, and over extended areas, to culminate in a short-distance docking state, where the outer membranes were separated by 2–3 nm. These large contact areas were devoid of protein densities and their periphery, where the distance between outer membranes reached 6–8 nm, was delimited by a ring-shaped macromolecular dense structure named the mitochondrial docking complex (MDC). Further GTP hydrolysis induced fusion of outer membranes at a single point of the MDC, where membrane curvature was most pronounced. Quantum dots labeling of Fzo1 demonstrated that it was enriched at the location of protein densities found in both long- and short-distance docking states, confirming that GTP hydrolysis by Fzo1 was responsible for the transition between the two states. Taken together, this work hints at a model in which cis- and trans-oligomerization of Fzo1, probably with the participation of co-factors such as Ugo1, results in formation of the MDC. The requirement of such macromolecular assembly for Mitofusin-mediated docking is further supported by the recent description of a hetero-oligomer formed between DLP1 and DLP2, two distinct DRPs from *Campylobacter jejuni* ^112^. These two bacterial DRPs were shown to assemble as a tetramer with two DLP2 units interacting together through their long four-helix bundle domain and two DLP1 units each interacting with one subunit of the DLP2 dimer. The organization of this tetramer strongly suggests its involvement in membrane docking processes and emphasizes the complexity with which DRPs can oligomerize. In this context, the discovery of the Fzo1-dependent MDC and the recent structural insights on MFN1 open up a range of fascinating possibilities regarding the precise molecular mechanisms underlying Mitofusin assembly.

## Mechanisms for mitochondrial fusion

Bringing membranes in close apposition is a necessary but not sufficient step for fusion to occur. Membranes must next be destabilized to allow the transition from bilayer to non-bilayer structures. Two recent studies have highlighted an important function of amphipathic helices of Mitofusin in triggering mitochondrial membrane fusion, possibly via lipid bilayer structure perturbation. In one study, the HR1 domain of MFN1 was shown to induce liposome fusion *in vitro* and to be required for MFN1-mediated mitochondrial fusion in cultured cells ^106^. The membrane fusion activity of HR1 was found to depend on a conserved amphipathic helix located at the C-terminal end of HR1. This amphipathic helix was shown to fold upon interaction with lipid bilayers, notably in regions presenting lipid packing defects (produced by either high local curvature or the presence of lipids with a cone-like molecular shape such as phosphatidylethanolamine [PE]). It was thus proposed that HR1 could mediate fusion by perturbing the lipid bilayer structure through a mechanism similar to that employed by the C-terminal tail of ATL in ER fusion. The parallel between the mechanisms driving ER and mitochondrial fusion was further highlighted in another study through the use of MFN1–ATL chimeras ^113^. When the TMD of MFN1 was replaced with that of ATL, the resulting chimera protein localized to ER tubules and could mediate ER fusion. In addition, an amphipathic helix (named α10) identified between the TMD and HR2 of MFN1 could functionally replace the C-terminal tail of ATL to mediate ER fusion *in situ* and liposome fusion *in vitro*. This amphipathic helix was also shown to interact with lipid bilayers and to display lipid-induced helical folding, notably in the presence of the mitochondrial lipids phosphatidylinositol (PI) and cardiolipin (CL).

The predicted GTP hydrolysis-induced conformational changes of MFN1 (either “scissor-like” or “self-folding” mechanisms) are expected to bring together the TMD of opposing Mitofusin proteins, along with the respective membranes in which they reside. This pulling mechanism might locally deform lipid bilayers around the TMD, allowing for the two neighboring amphipathic helices (HR1 and α10) to efficiently interact with, and perturb, the membrane structure. Close apposition of these two highly bent and destabilized membrane regions would result in spontaneous fusion ( Figure 3B). Local regulation of lipid bilayer composition, e.g. through the presence of cone-shaped lipids, can also produce bilayer packing defects similar to those generated by high membrane curvature ^114^. Interestingly, mitochondrial membranes contain close to 30 mol% of the conical lipid PE ^115^, and the phospholipase MitoPLD––which converts CL into the conical phosphatidic acid (PA) lipid––was shown to be required for mitochondrial fusion by acting downstream of Mitofusin-mediated docking ^98^. CL itself can adopt a conical shape upon binding to divalent cations like calcium or magnesium. In total, three different conical lipids of the outer mitochondrial membrane (PE, PA, and CL) could thus stimulate fusion by favoring membrane binding of the amphipathic helices HR1 and α10. Note that although CL is enriched in the inner mitochondrial membrane, its concentration can reach up to 20 mol% at the contact sites between inner and outer mitochondrial membranes ^115^, which is where fusion might take place ^76^. PE––as well as PA and CL (when they are bound to divalent cations)––are also known to induce the transition from lamellar (bilayer) to inverted hexagonal (non-bilayer) lipid phases ^116^, which is believed to occur during the formation of the stalk/hemifused fusion intermediate structure ^117^. Accordingly, all three lipids were found to stimulate protein-free liposome fusion *in vitro* ^118– 120^. The unsaturation status of lipid acyl chains can also induce profound changes on the mechanical properties of membranes ^121^. Interestingly, the ubiquitin–proteasome system was recently shown to modulate mitochondrial fusion by coordinating an intricate balance between the turnover of Fzo1 and the desaturation of fatty acids ^100^. Together, these results suggest that the last steps of mitochondrial fusion might be regulated by specific lipids that modify the structure of bilayers so as to facilitate their interaction with key protein sequences and/or their transition to non-bilayer fusion intermediate structures.

## Conclusion and perspectives

We are just beginning to understand the mode of action of Mitofusins in mitochondrial fusion. Recent biochemical and structural evidence indicates that Mitofusin-mediated mitochondrial fusion shares common mechanisms with ATL-mediated ER fusion. They both use GTP hydrolysis as a source of energy to induce membrane docking and amphipathic helices as the molecular trigger for fusion. It now remains to be determined whether and how the GTPase and HR domains of Mitofusins act synergistically during mitochondrial fusion. Future studies will also have to elucidate the precise molecular mechanisms underlying Mitofusin oligomerization and, notably, to clarify the involvement of lipid and/or protein co-factors in this process. The potential role for membrane contact sites in the regulation of mitochondrial fusion may also deserve particular attention. Contact sites between ER and mitochondria might allow the transfer of fusogenic lipids between these two organelles. Those between inner and outer mitochondrial membranes might also constitute privileged membrane regions, which are favorable for fusion (owing to their enrichment in cone-shaped fusogenic lipids) and which could further allow the functional coordination between the Mitofusins/Fzo and OPA1/Mgm1 fusion machineries ^76, 78, 79, 81, 82, 122^. The list of “things to be done” may expand with the elucidation of the modes of Mitofusin regulation by co-factors and post-translational modifications, but we can already expect that the coming decade will be as rich as the previous one in terms of discoveries in the field of mitochondrial fusion.
